# Association of remnant cholesterol with renal function and its progression in patients with type 2 diabetes related chronic kidney disease

**DOI:** 10.3389/fendo.2024.1331603

**Published:** 2024-07-04

**Authors:** Qiuhong Li, Tongdan Wang, Xian Shao, Xiaoguang Fan, Yao Lin, Zhuang Cui, Hongyan Liu, Saijun Zhou, Pei Yu

**Affiliations:** ^1^ NHC Key Laboratory of Hormones and Development, Chu Hsien-I Memorial Hospital and Tianjin Institute of Endocrinology, Tianjin Medical University, Tianjin, China; ^2^ Tianjin Key Laboratory of Metabolic Diseases, Tianjin Medical University, Tianjin, China; ^3^ Department of Nephrology, Fuwai Central China Cardiovascular Hospital, Zhengzhou, Henan, China; ^4^ Department of Epidemiology and Health Statistics, Tianjin Medical University, Tianjin, China

**Keywords:** remnant cholesterol, renal function, renal function progression, type 2 diabetes, chronic kidney disease

## Abstract

**Background:**

The association of Remnant cholesterol (RC) with renal function and its progression in patients with Type 2 diabetes (T2DM) related chronic kidney disease (CKD) remains unclear.

**Methods:**

8,678 patients with T2DM-related CKD were included in cross-sectional analysis, and 6,165 patients were enrolled in longitudinal analysis and followed up for a median of 36.0 months. The outcomes were renal composite endpoint event and rapid progression of renal function.

**Results:**

24.54% developed a renal composite endpoint event, and 27.64% rapid progression of renal function. RC levels above 0.56 mmol/L independently increased the risk of both renal composite endpoint (HR, 1.17; 95% CIs, 1.03-1.33) and rapid progression of renal function (OR, 1.17; 95% CIs, 1.01- 1.37). TG levels above 1.65 mmol/L only increased the risk of renal composite endpoint (HR, 1.16; 95% CIs, 1.02 -1.32). TC levels above 5.21 mmol/L increased the risk of renal composite endpoint (HR, 1.14; 95% CIs, 1.01-1.29) only in patients with proteinuria≥0.5g/d. Conversely, HDL-C levels below 1.20 mmol/L or above 1.84 mmol/L increased the risk of rapid progression of renal function (OR, 0.88; 95% CIs, 0.70 -0.99) in patients with proteinuria<0.5g/d (all P<0.05).

**Conclusion:**

In patients with T2DM-related CKD, RC was an independent risk factor for progression of renal function, and maintaining it below 0.56 mmol/L could reduce the risk of renal function progression.

## Background

Epidemiological research reveals that in 2021, over 537 million people globally had diabetes mellitus(DM), with 141 million in China (1, 2). The majority of cases were Type 2 diabetes(T2DM) (>95%) (3). Chronic kidney disease (CKD) is a significant cause of disability and mortality, posing a substantial global public health challenge (4, 5). Recent studies indicate that DM has surpassed glomerulonephritis as the primary cause of CKD in China (6). In 2019, DM-related CKD was responsible for 760,300 deaths, with T2DM accounting for 83.32% (7). Moreover, the annual cost of dialysis for CKD treatment alone amounts to a staggering 1.5 billion yuan (8). Therefore, early identification and intervention for high-risk individuals with renal function progression in T2DM-related CKD can significantly improve patient quality of life and alleviate the burden on the healthcare and economic systems.

Research has demonstrated a high prevalence of hyperlipidemia in individuals with DM, ranging from72-85% (9). Dyslipidemia is similarly prevalent in patients with CKD, attributed to notable changes significant alterations in lipoprotein metabolism, structure and function (10, 11). Consequently, individuals with T2DM-related CKD are more likely to be complicated by dyslipidemia (12). Studies indicate that hyperlipidemia can directly cause structural and functional changes in podocytes, mesangial cells and proximal tubular cells, ultimately leading to renal fibrosis and the progression of CKD (11, 13). Additionally, hyperlipidemia can indirectly induce glomerular and tubular damage by promoting pathological changes in mitochondria. Remnant cholesterol (RC) is the cholesterol of triglyceride-rich lipoproteins, which is composed of very low-density lipoprotein cholesterol (VLDL-C), intermediate-density lipoproteins (IDL), and postprandial chylomicrons (14). Studies have shown that RC is associated not only with atherosclerotic plaque formation (15), but also with inflammation (16, 17). The pathophysiological processes of glomerulosclerosis share similarities with those of atherosclerosis.

Currently, there exist inconsistencies in the relationship between blood lipid profiles and the progression of renal function, while the majority of research on RC in the kidney are cross-sectional studies examining its association with renal function (18–21). There is a lack of research investigating the association between RC and renal function, as well as its progression in patients with T2DM-related CKD through prospective studies. Therefore, the aim of this study was to investigate the relationship between RCand renal function through a cross-sectional analysis. Additionally, we aimed to explore the impact of RCon renal function progression through a prospective study, and compare with that of common lipid profiles, providing a foundation for lipid management in T2DM-related CKD patients to delay renal function progression.

## Methods

### Study populations

**Figure 1:**
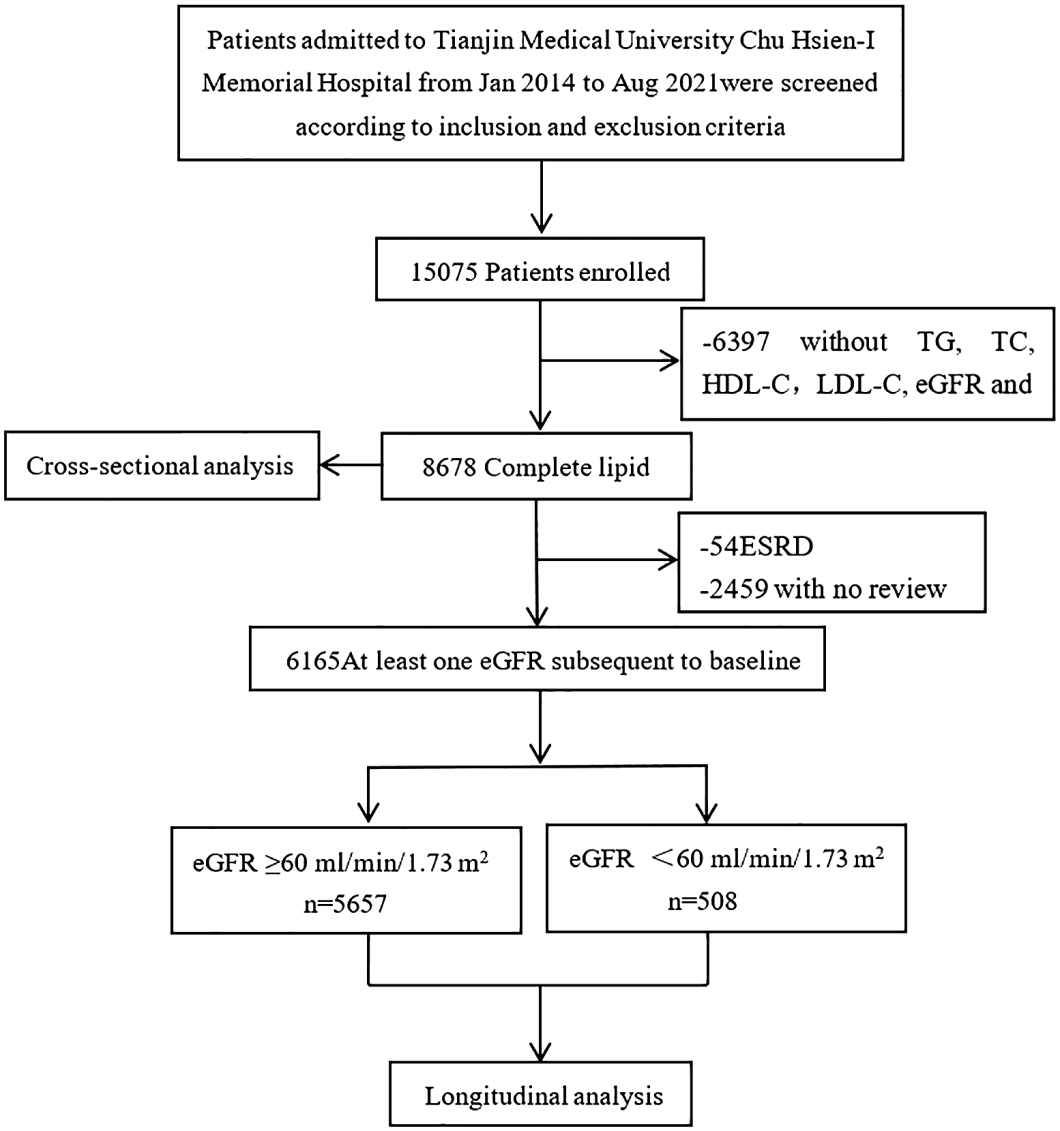
Flow chart of subject selection for this study.

Patients aged 18 and above with T2DM-related CKD were selected from the Chu Hsien-I Memorial Hospital of Tianjin Medical University between January 2014 and August 2021. The exclusion criteria used were: patients with (i) type 1 or other specific types of diabetes; (ii) acute kidney injury or end-stage renal disease; (iii) primary kidney disease or secondary kidney diseases caused by vasculitis, lupus, hypertension, or polycystic kidney disease; (iv) abnormalities in liver function tests (an alanine aminotransferase (ALT) or aspartate aminotransferase (AST) levels 2.5 times higher than normal), hepatitis B or hepatitis C infection; (v) endocrine metabolic diseases, such as Cushing’s syndrome or hyperthyroidism; (vi) complications, such as diabetic ketoacidosis and hyperosmolar hyperglycemia, hypoglycemia (fasting blood glucose<4 mmol/L), or other acute metabolic disorders; (vii) serious diseases, such as acute cardiovascular and cerebrovascular events, malignant tumors, hematological diseases, immune dysfunction, organ transplantation, or severe infections; (viii) using steroids and immunosuppressants within 6 months before enrollment; (ix) pregnancy or a malignancy. A total of 15,075 participants were initially enrolled in the study. After further screening, patients with missing data on triglycerides (TG), total cholesterol (TC), high-density lipoprotein cholesterol (HDL-C), low-density lipoprotein cholesterol (LDL-C), estimated glomerular filtration rate (eGFR), gender or other key information were excluded. The detailed screening procedure for participants is shown in **Figure 1**. The study was approved by the Ethics Committee of Tianjin Medical University Chu Hsien-I Memorial Hospital and adhered to the principles of the Helsinki Declaration of 1964.

### Data collection and laboratory assays

Height, weight, and blood pressure were obtained through medical examination. Weight in kilograms was divided by height squared in meters to calculate the body mass index (BMI). Information on the use of lipid-lowering drugs, ACEI/ARB antihypertensive drugs, sodium-glucose cotransporter-2 inhibitors (SGLT2i), and glucagon-like peptide-1 receptor agonists (GLP1RA) hypoglycemic agents was collected through consultations. Laboratory tests including white blood cell count (WBC), hemoglobin (HGB), platelet count (PLT), glycosylated hemoglobin (HbA1c), fasting blood glucose (FBG), liver function (AST, ALT), renal function (serum uric acid (SUA), blood urea nitrogen (BUN), and serum creatinine (SCR)), and lipid profiles (LDL-C, HDL-C, TG, TC), 24-hours total urine protein (24hTP), and urinary albumin-to-creatinine ratio (UACR) were conducted through laboratory tests. Blood samples were collected in the morning after an 8-hour fasting period, while urine samples were collected in the early morning and 24-hours the day before. At the Clinical Laboratory Center of Tianjin Medical University Chu Hsien-I Memorial Hospital, all samples were performed. The eGFR (mL/min/1.73m²) was calculated using chronic kidney disease epidemiology collaboration (CKD-EPI) formula. The RC was calculated using the formula: RC (mmol/L) = TC (mmol/L) - HDL-C(mmol/L) - LDL-C(mmol/L) (22, 23).

### Definition of covariates

The renal composite endpoint event was defined as a 30% decrease in eGFR from baseline, progression to eGFR<60ml/min/1.73m^2^ (for patients with eGFR≥60mL/min/1.73m^2^ at baseline) or ESRD (eGFR<15ml/min/1.73m^2^). Rapid progression of renal function was defined as a mean annual decline in eGFR>5ml/min/1.73m^2^.

### Statistical analysis

Baseline data for continuous variables are presented as mean ± standard deviation (SD) if they were approximately normally distributed and median and interquartile range (IQR) if they were skewed. Categorical variables were presented as a number (percentage). Baseline characteristics were compared among groups stratified by eGFR levels using ANOVA or Kruskal-Wallis tests for continuous variables, and chi-square (χ^2^) tests for categorical variables. An analysis of the relationship between lipid profiles, including RC, and eGFR was carried out using a multivariate linear regression model. The association between lipid profiles and renal composite endpoint events was examined using Cox proportional hazards regression models (results presented as hazard ratios (HRs) and 95% confidence interval (95% CI)). The relationship between lipid profiles and rapid progression of renal function was analyzed using logistic regression model (results presented as odds ratios (ORs) and 95% CI). The dose-response relationship between lipid profiles and renal function progression was analyzed using restricted cubic spline (RCS) curves. Furthermore, we conducted subgroup analyzes to identify potential factors that could impact the stability of the findings.

Statistical analyses were performed using SPSS 24.0 software (SPSS Inc., Chicago, IL, USA), R version 4.2.1 (www.r-project.org) and GraphPad Prism version 8.0 software (San Diego, CA, USA). P-value<0.05 were considered statistically significant.

## Results

### Baseline characteristics

A total of 8,678 patients were included in the cross-sectional study. Clinical characteristics stratified by baseline eGFR level are presented in **Table 1**. The mean age was 59.56 ± 9.97 years, including 4,970 males (57.27%, age 58.20 ± 10.51 years) and 3,708 females (42.73%, age 61.38 ± 8.88 years). Patients with lower eGFR had a higher SBP, WBC, ACR, 24hTP, BUN, SCR, SUA, RC and TG, and lower HGB, FBG, ALT, AST, HDL-C, SGLT2i and GLP-1RA hypoglycemic drug use. The differences were significant (P<0.05).

**Table 1:** Baseline characteristics of the populations stratified by baseline eGFR levels.

Characteristic	eGFR group					P value
	All	Stage1 (eGFR≥90ml/min/1.73m^2^)	Stage2 (eGFR 60~90ml/min/1.73m^2^)	Stage3 (eGFR30~60ml/min/1.73 m^2^)	stage4~5 (eGFR<30ml/min/1.73 m^2^)	
N	8678	5277	2503	755	143	
Demographics						
Male, n (%)	4970(57.3)	2397(55.7)	1531(61.2)	417(55.2)	85(59.4)	0.000
Age(y)	59.56 ± 9.97	57.47 ± 9.73	62.38 ± 9.14	64. 37 ± 10.03	61.69 ± 10.88	0.000
Physical characteristics						
SBP (mmHg)	140.34 ± 18.70	139.02 ± 17.64	141.18 ± 19.45	144.37 ± 21.10	149.59 ± 21.01	0.000
BMI (kg/m^2^)	26.88(24.62-29.4)	26.89(24.61-29.58)	27.05(24.77-29.40)	26.59(24.69-29.05)	25.78(23.58-28.42)	0.273
Biochemical measurements						
WBC(*10^9^/L)	6.90 ± 1.78	6.78 ± 1.74	6.95 ± 1.75	7.40 ± 1.89	7.83 ± 2.31	0.000
HGB(g/L)	141.13 ± 18.06	143.80 ± 15.92	141.20 ± 17.86	128.09 ± 19.72	103.45 ± 21.28	0.000
PLT(*10^9^/L)	219.00(184.00-260.00)	219.00(185.00-260.00)	217.00(183.00-257.00)	221.50(185.25-267.00)	223.00(186.00-260.50)	0.304
ACR (mg/g)	162.69(62.74-386.02)	110.84(53.27-260.84)	204.07(76.81-418.63)	316.50(86.61-569.65)	564.25(453.32-769.80)	0.000
24hTP(g/d)	0. 30(0.14-1.03)	0.24(0.14-0.60)	0. 38(0.15-1.35)	1.10(0.19-2.70)	2.28(1.08-3.64)	0.000
FBG (mmol/L)	9.16(7.65-11.58)	9.39(7.83-11.98)	8.87(7.52-11.11)	8.51(7.14-11.15)	7.40(5.90-9.42)	0.000
AST(U/L)	17.90(14.60-22.3)	18.00(14.70-22.70)	18.10(14.90-22.30)	16.90(13.88-20.80)	15.10(12.20-17.90)	0.000
ALT(U/L)	18.80(13.60-27.1)	20.10(14.50-29.20)	18.60(13.70-26. 20)	15.05(11.03-20.80)	11.90(8.85-16.45)	0.000
BUN (mmol/L)	6.32 ± 2.71	5.45 ± 1.34	6.53 ± 1.57	9.30 ± 2.59	18.92 ± 8.18	0.000
SCR (umol/L)	78.09 ± 54.57	60.08 ± 11.51	84.39 ± 13.79	125.94 ± 26.32	379.69 ± 231.29	0.000
eGFR (ml/min/1.73m^2^)	97.52 ± 30.41	116.27 ± 20.38	77.35 ± 8.35	48.48 ± 8.30	17.85 ± 7.83	0.000
HbA1c (%)	8.41 ± 1.82	8.52 ± 1.81	8.23 ± 1.80	8.37 ± 1.81	7.59 ± 1.66	0.000
SUA (umol/L)	456.60 ± 113.22	308.24 ± 81.97	352.37 ± 87.45	407.37 ± 101.12	456.60 ± 113.22	0.000
RC (mmol/L)	0.55(0.39-0.80)	0.53(0.38-0.80)	0.55(0.40-0.80)	0.63(0.44-0.91)	0.63(0.47-0.86)	0.000
TG (mmol/L)	1.62(1.16-2.40)	1.5(1.12-2.37)	1.63(1.19-2.34)	1.81(1.28-2.75)	1.91(1.24-2.51)	0.000
TC (mmol/L)	5.23 ± 1.22	5.19 ± 1.16	5.24 ± 1.23	5.41 ± 1.50	5.27 ± 1.61	0.001
LDL-C(mmol/L)	3.34 ± 0.99	3.31 ± 0.93	3.37 ± 0.99	3.50 ± 1.21	3.44 ± 1.35	0.000
HDL-C(mmol/L)	1.20(1.04-1.40)	1.20(1.08-1.40)	1.20(1.02-1.40)	1.19(1.00-1.37)	1.10(0.90-1.29)	0.000
Using of drugs						
Hypolipidemic drugs, n (%)	1048(12.1)	632(12)	304(12.1)	95(12.6)	17(11.9)	0.966
SGLT2i, n (%)	436(5)	307(5.8)	115 (4.6)	14 (1.9)	0(0)	0.000
GLP-1RA, n (%)	272(3.1)	186(3.5)	76(3)	10(1.3)	0(0)	0.000
ACEI/ARB, n (%)	1190(13.7)	643(12.2)	396(15.8)	137(18.1)	14(9.8)	0.000

eGFR, estimated glomerular filtration rate; SBP, systolic blood pressure; BMI, body mass index; WBC, white blood cell count; HGB, hemoglobin; PLT, platelet count; ACR, albumin-to-creatinine ratio; 24hTP, 24-hours total urine protein; FBG, fasting blood glucose; ALT, alanine aminotransferase; AST, aspartate aminotransferase; BUN, blood urea nitrogen; SCR, serum creatinine; HbA1c, glycated hemoglobin; SUA, serum uric acid; RC, remnant cholesterol; TG, plasma triglyceride level; TC, total cholesterol; LDL-C, low-density lipoprotein cholesterol; HDL-C, high-density lipoprotein cholesterol; and SGLT2i, sodium-glucose cotransporter-2 inhibitors; GLP1RA, glucagon-like peptide-1 receptor agonists.

Data are presented as the mean ± standard error or median (interquartile range) for continuous variables, and the numbers (percentage) for categorical variables.

P < 0.05 was considered statistically significant.

### Relationship between lipid profiles, including RC, and baseline eGFR

**Table 2** reveals the relationship between lipid profiles, including RC, and baseline eGFR. The results showed that each SD increase in RC, TC and LDL-C was significantly associated with a lower baseline eGFR (β=-1.48, β=-1.17, β=-1.59, respectively), while each SD increase in HDL-C was associated with a higher baseline eGFR (β=2.03) (all P<0.05). After adjusting for confounding factors, baseline eGFR was still significantly correlated with RC (β=-2.51, P=0.001), TC (β=-2.12, P=0.004), LDL-C (β=-1.89, P=0.007) and HDL-C (β=1.86, P=0.022).

**Table 2:** The relationship between lipid profiles and the baseline eGFR.

Lipoprotein	Unadjusted		Adjusted	
	β coefficient of Lipoprotein Variable (95% CI)	P value	β coefficient of Lipoprotein Variable (95% CI)	P value
RC (mmol/L)	-1.48(-2.30to-0.66)	0.000	-2.51(-3.98to-1.03)	0.001
TC (mmol/L)	-1.17(-1.84to-0.50)	0.001	-2.12(-3.55to-0.68)	0.004
TG (mmol/L)	-0.30(-1.13to0.53)	0.477	-1.16(-2.63to0.30)	0.120
LDL-C (mmol/L)	-1.59(-2.24to-0.94)	0.000	-1.89(-3.27to-0.51)	0.007
HDL-C (mmol/L)	2.03(1.39to2.67)	0.000	1.86(0.27to3.45)	0.022

CI, confidence interval.

The β estimate is the difference in eGFR (ml/min per 1.73 m^2^ per year) per 1 SD increase in lipoprotein variable.

Unadjusted: no adjustment.

Adjusted: adjusted for age, gender,24hTP, BMI, SBP, HbA1c, HGB, ACEI/ARB, SGLT2i, GLP-1RA and Lipid-lowering agents use at baseline.

### Relationship between lipid profiles, including RC, and the progression of renal function

A total of 6,165 patients with at least one recorded follow-up of eGFR were included in the prospective analysis. At a median follow-up of 36 months, 1,513 patients (24.54%) reached a renal composite endpoint event, while 1,704 patients (27.64%) showed rapid progression of renal function event.

**Table 3** presents the association between lipid profiles and the risk of renal composite endpoint event. Although elevated RC, TC, TG, LDL-C and HDL-C levels were all associated with the risk of renal composite endpoint event in the unadjusted model (all P<0.05). After adjusting for confounding factors, only RC, TC and TG could independently predict the renal composite endpoint event, with HRs (95% CIs) of 1.17 (1.03, 1.33),1.14 (1.01, 1.29), and 1.16 (1.02, 1.32), respectively (all P<0.05).

**Table 3:** The relationship between lipid profiles and the renal composite endpoint event.

Lipoprotein	Unadjusted		Adjusted	
	HR per 1 SD increase of Lipoprotein Variable (95% CI)	P value	HR per 1 SD increase of Lipoprotein Variable (95% CI)	P value
RC (mmol/L)	1.27(1.20-1.34)	0.000	1.17(1.03-1.33)	0.013
TC (mmol/L)	1.12(1.06-1.18)	0.000	1.14(1.01-1.29)	0.033
TG (mmol/L)	1.24(1.17-1.30)	0.000	1.16(1.02-1.32)	0.024
LDL-C (mmol/L)	1.09(1.04-1.15)	0.000	1.09(0.97-1.23)	0.135
HDL-C (mmol/L)	0.87(0.83-0.92)	0.000	1.03(0.90-1.19)	0.659

1SD, 1 standard deviation; CI, confidence interval; HR, hazard ratios.

Unadjusted: no adjustment.

Adjusted: adjusted for age, gender, baseline eGFR,24TP, BMI, SBP, HbA1c, HGB, ACEI/ARB, SGLT2i, GLP-1RA and Lipid-lowering agents use at baseline.

**Table 4** shows the association between lipid profiles and the risk of rapid progression of renal function. After adjusting for covariates, only RC and HDL-C could independently predict the occurrence of rapid progression of renal function, with ORs (95% CIs) of 1.17 (1.01, 1.37) and 0.88 (0.70, 0.99), respectively (P<0.05).

**Table 4:** The relationship between lipid profiles and the rapid progression of renal function.

Lipoprotein	Unadjusted		Adjusted	
	OR per 1 SD increase of Lipoprotein Variable (95% CI)	P value	OR per 1 SD increase of Lipoprotein Variable (95% CI)	P value
RC (mmol/L)	1.16(1.08-1.24)	0.000	1.17(1.01-1.37)	0.048
TC (mmol/L)	1.06(1.00-1.12)	0.058	1.02(0.88-1.19)	0.792
TG (mmol/L)	1.16(1.08-1.24)	0.000	1.16(1.00-1.36)	0.057
LDL-C (mmol/L)	1.07(1.01-1.13)	0.000	1.00(0.86-1.15)	0.942
HDL-C (mmol/L)	0.86(0.81-0.91)	0.000	0.88(0.70-0.99)	0.047

1SD, 1 standard deviation; CI, confidence interval; OR, odds ratios.

Unadjusted: no adjustment.

Adjusted: adjusted for age, gender, baseline eGFR,24TP, BMI, SBP, HbA1c, HGB, ACEI/ARB, SGLT2i, GLP-1RA and Lipid-lowering agents use at baseline.

### Dose-response relationship of blood lipid profiles and the risk of progression of renal function

**Figure 2:**
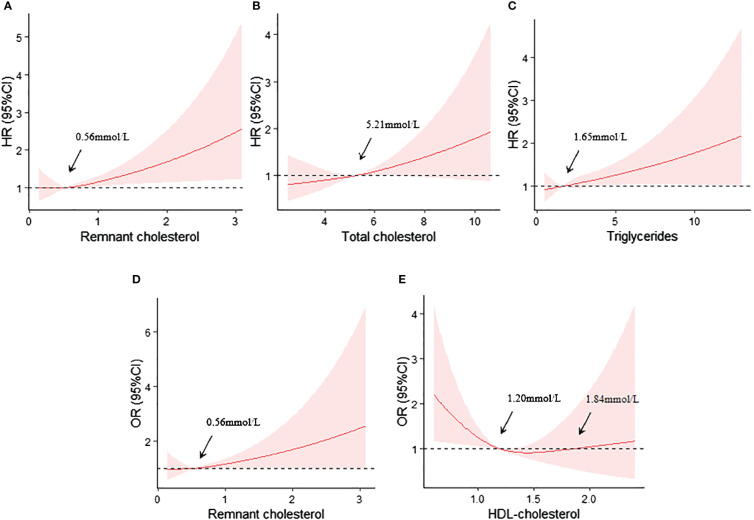
**(A)** Restricted cubic spline curves analyses of RC and the renal composite endpoint event; **(B)** Restricted cubic spline curves analyses of TC and the renal composite endpoint event; **(C)** Restricted cubic spline curves analyses of TG and the renal composite endpoint event; **(D)** Restricted cubic spline curves analyses of RC and the rapid progression of renal function; **(E)** Restricted cubic spline curves analyses of HDL-C and the rapid progression of renal function. All adjusted for age, gender, baseline eGFR,24TP, BMI, SBP, HbA1c, HGB, ACEI/ARB, SGLT2i, GLP-1RA and Lipid-lowering agents use at baseline.

The RCS curve analyses revealed that RC, TC and TG were linearly positively correlated with the risk of renal composite endpoint event (nonlinear P>0.05) after controlling for confounders, and the cut-off points were 0.56mmol/L, 5.21mmol/L and 1.65mmol/L, respectively (**Figures 2A–C**, respectively).

Similarly, RC also exhibited a linearly positively correlation with the risk of rapid progression of renal function (nonlinear P>0.05), with a cut-off point of approximately 0.56mmol/L (**Figure 2D**). However, HDL-C level showed a U-shaped relationship with the risk of rapid progression of renal function. The cut-off points were 1.20mmol and 1.84mmol (**Figure 2E**).

### Subgroup analyses

**Figure 3:**
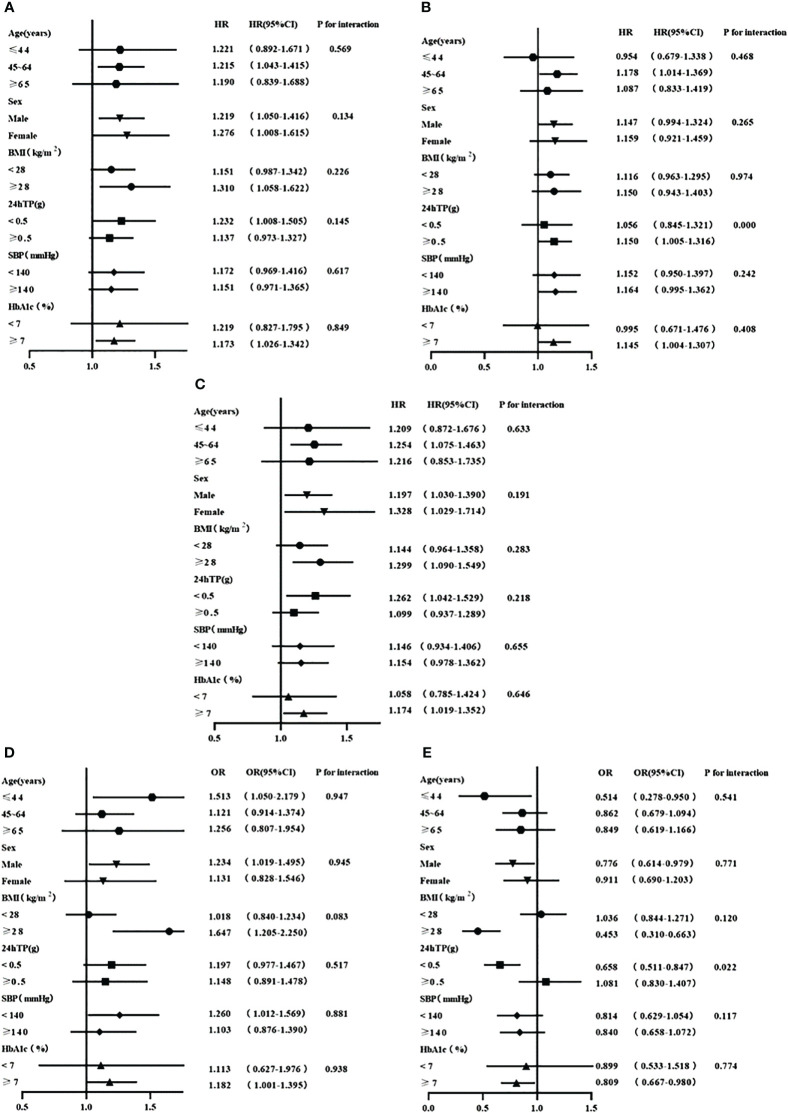
**(A)** Stratified analyses of the relationship between RC and the renal composite endpoint event; **(B)** Stratified analyses of the relationship between TC and the renal composite endpoint event; **(C)** Stratified analyses of the relationship between TG and the renal composite endpoint event; **(D)** Stratified analyses of the relationship between RC and the rapid progression of renal function; **(E)** Stratified analyses of the relationship between HDL-C and the rapid progression of renal function. All adjusted for age, gender, baseline eGFR,24TP, BMI, SBP, HbA1c, HGB, ACEI/ARB, SGLT2i, GLP-1RA and Lipid-lowering agents use at baseline.

We further explored the relationships between the lipid profiles that were independently associated with the progression of renal function and corresponding renal outcomes, stratified by age, sex, BMI, 24hTP, SBP and HbA1c. No significant heterogeneity was observed (all P for interaction>0.05) in the relationship between RC and the risk of renal composite endpoint event and rapid progression of renal function (**Figures 3A, D**). Similarly, the relationship between TG and the risk of the renal composite endpoint was stable across all subgroups (**Figure 3C**).

In the subgroup analysis examining the association between TC and the risk of renal composite endpoint event, we found that TC level was associated with the risk of a renal composite endpoint only among patients with proteinuria≥0.5g/d (P=0.000 for interaction, HR 95%CI, 1.150 (1.005, 1.316)) (**Figure 3B**). However, the association between HDL-C and the risk of rapid progression of renal function existed only in patients with proteinuria<0.5g/d (P=0.022 for interaction, HR 95%CI, 0.658 (0.511, 0.847)) (**Figure 3E**).

## Discussion

In patients with T2DM-related CKD, our study not only found that RC, TC, and LDL-C were negatively correlated with baseline eGFR, while HDL-C was positively correlated with baseline eGFR, but also demonstrated an independent association between RC and the risk of renal composite endpoint event as well as rapid progression of renal function. TG was found to be associated with the risk of renal composite endpoint event. The relationships observed were linear and robust. Additionally, our results showed significant linear relationship between TC and the risk of renal composite endpoint event only among patients with proteinuria≥0.5g/d, while a U-shape relationship between HDL-C and the risk of rapid progression of renal function only in patients with proteinuria<0.5g/d in the prospective cohort analysis. More importantly, our study revealed the optimal management value of lipid profiles to prevent the progression of renal function.

The negative association between RC and baseline eGFR is supported by previous studies conducted on patients with non-diabetic related CKD (21). One explanation may be that RC is cholesterol contained in triglyceride-rich lipoproteins. Studies have shown that the production of triglyceride-rich lipoproteins is increased and the clearance is decreased in CKD patients (24). Another explanation may be its composition, as studies have revealed that VLDL clearance from the circulation and conversion to IDL is also impaired in CKD patients due to reduced expression of VLDL receptors in adipocytes and muscle cells (25). Moreover, the metabolism, structure and post-translational modification of postprandial lipoproteins and triglyceride-rich lipoproteins are changed in CKD patients (26). In addition, the increased production and decreased metabolism of chylomicrons due to insulin resistance in T2DM may also contribute to the elevated RC levels (27). However, further in-depth studies are needed to explore the specific mechanisms underlying RC increase in T2DM-related CKD.

Our study provides novel evidence that elevated RC levels were associated with an increased risk of renal composite endpoint event and rapid progression of renal function in T2DM-related CKD patients. The mechanism underlying this relationship is not fully understood. It may be that elevated RC concentrations not only contribute to atherosclerosis similar to LDL-C (28, 29), but also promote systemic low-grade inflammation (30). Furthermore, studies have shown that RC can enhance the expression of adhesion molecules, coagulation factors, and inflammatory proteins in endothelial cells, leading to monocyte recruitment and attachment, and promote foam cell formation (31). Animal studies has also demonstrated a link between atherogenic lipid profiles and endothelial dysfunction and glomerulosclerosis (32).

We found that TG was associated with the risk of renal composite endpoint event in patients with T2DM-related CKD, while the relationship between TG levels and renal function progression in previous studies are inconsistent (33–35). Comparative analysis suggests that in patients with DM or massive proteinuria, TG may have a certain correlation with renal function, whereas dyslipidemia alone may not be sufficient to cause renal damage in individuals without DM or massive proteinuria. However, in patients with DM or massive proteinuria, typical metabolic disorders may contribute to the lipotoxic effects on the microvascular bed, which are necessary for the progression of renal function (34).

Consistent with previous studies (36, 37), our findings indicate that low levels of HDL-C increase the risk of rapid progression of kidney function in patients with T2DM-related CKD. However, no correlation was found between HDL-C and progression of renal function in some studies, which were carried out in populations with an overall eGFR lower than 60mL/min/1.73m^2^ (36, 38). The reason maybe that the protective functions of HDL-C affected due to toxins, inflammation and other environmental factors in these populations (39). Studies have also demonstrated that under conditions of inflammation and oxidative stress, HDL-C may not only be dysfunctional, but can also convert antioxidant and anti-inflammatory substances into pro-oxidant and pro-inflammatory particles (40, 41).

The most clinically relevant finding in our study was that the risk of renal function progression was significantly increased in T2DM-related CKD patients when RC, TC and TG levels exceeded 0.56mmol/L, 5.21mmol/L and 1.65mmol/L, respectively, and these cut-off values align with the lipid level control targets recommended in the Chinese guidelines for diagnosis and treatment of diabetic kidney disease (2021) (42). Additionally, our study revealed a U-shaped relationship between HDL-C and rapid progression of renal function in T2DM-related CKD patients, which was consistent with previous studies conducted on kidney and heart disease (37, 43). When HDL-C levels were lower than 1.20mmol/L or higher than 1.84mmol/L, the risk of rapid progression of renal function increased. The reason may be that although HDL-C has anti-oxidation, anti-inflammation and anti-thrombosis effects (44, 45), high HDL-C levels paradoxically promote senescence, weaken endothelial progenitor cell tube formation, and impair angiogenesis (46).

Subgroup analysis showed that HDL-C loses its protective effect on rapid progression of renal function in patients with proteinuria≥0.5g/d. This may be due to the fact that the protective effect of HDL-C depends not only on its circulating concentration but also on the “qualitative” characteristics of HDL-C particles (47). Increased concentration of advanced glycation end products in patients with diabetes and kidney disease impairs the antioxidant capacity of HDL-C (48). Similar to the interaction between TC and urine protein found in previous studies (36), our research also found that TC levels were associated with the risk of renal composite endpoint event only in patients with proteinuria≥0.5g/d. It may be that proteinuria not only causes dyslipidemia, but also promotes the progression of kidney disease (49, 50), and that this interaction makes the relationship between TC and the occurrence of renal function progression more obvious in individuals with high proteinuria concentrations.

Our study has several strengths. Firstly, we explored the association of lipid profiles, especially RC, and renal function as well as its progression in T2DM-related CKD patients for the first time using a comprehensive design that includes cross-sectional and prospective analysis. Furthermore, we revealed the optimal lipid profile management value. Secondly, we adjusted for potential confounders and performed a robust analysis.

However, there are some limitations to acknowledge. Firstly, RC values were calculated rather than directly measured, and there may be some deviations between the actual and calculated values. Nevertheless, calculated RC values are valid and have been widely used in large Danish cohort studies (22, 23). Secondly, study populations were T2DM-related CKD patients, and the application of the findings of this study to other populations needs further validation. Thirdly, this is an observational study and as such, the causal relationship between the lipid profiles and progression of renal function could not be confirmed.

## Conclusions

In summary, in T2DM-related CKD populations, RC, TC and LDL-C levels were negatively correlated with renal function, while HDL-C levels were positively correlated with renal function. However, only RC was an independent risk factor for both renal composite endpoint event and rapid progression of renal function event. Maintaining RC levels below 0.56mmol/L can reduce the risk of progression of renal function. Further research is needed to validate these results in other cohorts and elucidate the underlying mechanisms driving the relationship.

## Data availability statement

The original contributions presented in the study are included in the article/supplementary material, further inquiries can be directed to the corresponding author/s.

## Ethics statement

The studies involving humans were approved by the ethics committee of Tianjin Chu Hsien-l Memorial Hospital. The studies were conducted in accordance with the local legislation and institutional requirements. The ethics committee/institutional review board waived the requirement of written informed consent for participation from the participants or the participants’ legal guardians/next of kin because the data were anonymous and observational.

## Author contributions

QL: Data curation, Formal analysis, Methodology, Writing – original draft, Writing – review & editing. TW: Formal analysis, Writing – review & editing. XS: Formal analysis, Writing – review & editing. XF: Writing – review & editing. YL: Writing – review & editing. ZC: Formal analysis, Writing – review & editing. HL: Writing – review & editing. SZ: Writing – review & editing. PY: Formal analysis, Funding acquisition, Methodology, Writing – review & editing.
